# Limbic Responses Following Shock Wave Exposure in Male and Female Mice

**DOI:** 10.3389/fnbeh.2022.863195

**Published:** 2022-06-07

**Authors:** Eileen H. McNamara, Laura B. Tucker, Jiong Liu, Amanda H. Fu, Yeonho Kim, Patricia A. Vu, Joseph T. McCabe

**Affiliations:** ^1^Department of Anatomy, Physiology and Genetics, Uniformed Services University of the Health Sciences, Bethesda, MD, United States; ^2^Pre-Clinical Studies Core, Center for Neuroscience and Regenerative Medicine, Henry M. Jackson Foundation, Bethesda, MD, United States

**Keywords:** anxiety, blood-brain barrier, c-Fos, depression, limbic, post-traumatic stress disorder, blast traumatic brain injury (bTBI), righting reflex

## Abstract

Blast traumatic brain injury (bTBI) presents a serious threat to military personnel and often results in psychiatric conditions related to limbic system dysfunction. In this study, the functional outcomes for anxiety- and depressive-like behaviors and neuronal activation were evaluated in male and female mice after exposure to an Advanced Blast Simulator (ABS) shock wave. Mice were placed in a ventrally exposed orientation inside of the ABS test section and received primary and tertiary shock wave insults of approximately 15 psi peak pressure. Evans blue staining indicated cases of blood-brain barrier breach in the superficial cerebral cortex four, but not 24 h after blast, but the severity was variable. Behavioral testing with the elevated plus maze (EPM) or elevated zero maze (EZM), sucrose preference test (SPT), and tail suspension test (TST) or forced swim test (FST) were conducted 8 days–3.5 weeks after shock wave exposure. There was a sex difference, but no injury effect, for distance travelled in the EZM where female mice travelled significantly farther than males. The SPT and FST did not indicate group differences; however, injured mice were less immobile than sham mice during the TST; possibly indicating more agitated behavior. In a separate cohort of animals, the expression of the immediate early gene, c-Fos, was detected 4 h after undergoing bTBI or sham procedures. No differences in c-Fos expression were found in the cerebral cortex, but female mice in general displayed enhanced c-Fos activation in the paraventricular nucleus of the thalamus (PVT) compared to male mice. In the amygdala, more c-Fos-positive cells were observed in injured animals compared to sham mice. The observed sex differences in the PVT and c-Fos activation in the amygdala may correlate with the reported hyperactivity of females post-injury. This study demonstrates, albeit with mild effects, behavioral and neuronal activation correlates in female rodents after blast injury that could be relevant to the incidence of increased post-traumatic stress disorder in women.

## Introduction

Blast exposure is the leading cause of traumatic brain injury (TBI) in military personnel and a serious threat to civilian populations in proximity to regional conflicts and civil unrest. Blast TBI (bTBI) is considered the “invisible wound” in modern day combat zones, such as Iraq and Afghanistan, and milder injury with ∼80% prevalence is the most common form. Blast exposure has been associated with a variety of psychiatric conditions, including post-traumatic stress disorder (PTSD), depression, and anxiety (Walker et al., 2015). Post-mortem examination of chronic bTBI cases also found histories of enduring neuropsychiatric symptoms (Rosenfeld and Ford, 2010; Shively et al., 2016; Mac Donald et al., 2017; Ryan-Gonzalez et al., 2019).

Preclinical reports have shown central nervous system (CNS) limbic system areas are particularly vulnerable to bTBI, and are often associated with neurobehavioral changes related to anxiety, depression, and PTSD (Elder et al., 2012; Blaze et al., 2020; Kostelnik et al., 2021). Alterations in the basolateral amygdala (BLA) have been reported following bTBI with changes to neuronal cytostructure, gene expression, and neuroimmune responses with associated anxiety-like behavior (Sajja et al., 2015; Ratliff et al., 2019; Blaze et al., 2020). Likewise, blast injury in rats altered a marker associated with PTSD, stathmin 1, in the amygdala, but not in the hippocampus (Elder et al., 2012). However, ultrastructural rat hippocampal changes have been reported after blast (Cernak et al., 2001). The paraventricular nucleus of the thalamus (PVT) is another important area for emotion-based responses, especially fear. The PVT is primarily involved in stress, arousal, and motivated behaviors with projections to the amygdala and limbic cortex (Kirouac, 2015; Azevedo et al., 2020; Rowson and Pleil, 2021), and it was recently found to mediate PVT-central amygdala freezing responses (Ma et al., 2021). The PVT’s connection to depressive-like behavior is less understood, but reduced tail suspension test immobility with PVT inhibition has been reported (Kato et al., 2019; Barson et al., 2020). Other cortical changes after bTBI include a decrease in Thy-1 stained cortical neuronal afferents, possibly from the medial prefrontal cortex, which terminate in the BLA (Heldt et al., 2014). These limbic regions are also involved in fear conditioning; a common preclinical model for evaluating PTSD. Blast exposure increased responses in the acoustic startle reflex and anxiety-related behaviors in the elevated plus and zero mazes (Xie et al., 2013; Awwad et al., 2015). Preclinical and clinical imaging data following blast exposure correspondingly indicate greater amygdala activation and long-term anxiety post-bTBI, including region-specific imaging differences in brain metabolism in the amygdala and blood-brain barrier (Matthews et al., 2011; Rubovitch et al., 2011; Jaiswal et al., 2019).

Acute immediate early gene (IEG) responses in the cerebrum are observed after a broad range of stimuli including cell insults, immune activation, apoptosis, neuronal depolarization, and learning and memory experiences (Curran and Morgan, 1995; Raghupathi et al., 1995; Chaudhuri, 1997; Gallo et al., 2018). Alterations in c-Fos expression may play a role in encoding transient stimuli to long-term genetic changes. A marker of neuronal activation, c-Fos expression becomes elevated in the PVT after stressors such as the forced swim test and elevated plus maze (Curran and Morgan, 1995; Gallo et al., 2018; Barson et al., 2020). Changes in c-Fos have been reported as early as 1–3 h after blast exposure in the hippocampus and amygdala (Säljö et al., 2002; Du et al., 2013; Rex et al., 2013; Ou et al., 2022), and elevated levels can persist (Säljö et al., 2002; Russell et al., 2018b). As described earlier, the PVT and central amygdala are involved in fear conditioning, and increased c-Fos activation in these regions was reported in a single prolonged stress mouse model of PTSD (Penzo et al., 2015; Park and Chung, 2019; Azevedo et al., 2020). Only one study has examined IEG response 7 days after a restraint stressor and bTBI, and found sex differences in c-Fos response in the paraventricular nucleus of the hypothalamus (Russell et al., 2018b). Sex differences in functional outcome are also observed with more activity displayed by females compared to males after TBI (Tucker et al., 2016, 2017). One group described increased risk behavior in males in response to negative outcomes, lack of reward during a gambling task with the choice to select a more secure option, compared to female rats (Ishii et al., 2018).

The objective of this exploratory study was to determine how a shock wave exposure alters physiological and behavioral outcomes. To assess the effects of primary and tertiary shock wave injury, the Advanced Blast Simulator (ABS) was utilized as a reliable state-of-the-art model of bTBI (Sawyer et al., 2016). The elevated plus and zero maze (EPM, EZM), sucrose preference test (SPT), tail suspension test (TST), and forced swim test (FST) were performed to assess anxiety- and depressive-like behaviors after ABS, while blood-brain barrier (BBB) and c-Fos neuronal activation were studied for acute pathological changes.

## Materials and Methods

### Animals

Eight-week old male and cycling female C57BL/6J mice (00664) were obtained from the Jackson Laboratory (Bar Harbor, ME, United States) and housed in an AAALAC-accredited animal facility for at least 3 days of acclimation before the experiments were started. All procedures were approved by the Uniformed Services University of the Health Sciences (USUHS) Institutional Animal Care and Use Committee. Until the SPT was conducted, all animals were group-housed (five per cage), had access to food (Harlan Teklad Global Diets 2018, 18% protein) and water *ad libitum*, and were maintained on a standard 12 h: 12 h light-dark cycle. Animals that underwent the SPT were divided into separate cages with standard enrichment (cotton nestlets and huts) for the duration of the study, but singly housed mice were still able to view neighboring cages. All experimental procedures were performed by female investigators.

### Advanced Blast Simulator

Male and female mice were randomly assigned to injured or sham conditions. Injured groups were exposed to a single blast overpressure ∼ 15 psi peak pressure using the USUHS Advanced Blast Simulator (ABS) as previously described (Vu et al., 2018). Briefly, the ABS contains a driver and driven chamber with pressure transducers placed within the inner wall to monitor incident pressure and shock wave velocity. A pencil gauge probe immediately adjacent to the mouse holder measured incident pressure (Quartz, free-field, ICP blast pressure pencil probe, 50 psi, 104.2 mV/psi, 137B23A, PCB Piezotronics). A membrane consisting of two or three 0.254-mm thick clear acetate sheets (Grafix Plastics, Cleveland, OH, United States) and two layers of vinyl-coated polyester mesh (Pet Screen, Hanover/New York Wire, Cat. No. 70589, mesh size: 14.5 × 10 grids/in^2^, wire diameter: 0.635 mm) separated the driver and the driven chambers. Sham-treated mice were anesthetized and placed near the blast chamber, but were not exposed to the blast wave. All mice were first placed in an isoflurane induction chamber (3% isoflurane in 100% oxygen for 4–6 min) and once anesthetized, head and body wraps (a modified tongue depressor and Vet Wrap) were used to minimize movement before the mouse was placed inside the simulator. A hatch in the driven chamber allowed the ABS mouse to be secured in a mesh holder (same material as the membrane, 14cm × 15cm with a cross-sectional areal occlusion of ∼5.6% for the shock wave) supported by metal posts (12.7 mm diameter, about 2.9 m distal to the driver membrane) in a vertical orientation, exposing the ventral surface of the mouse to the oncoming blast wave. Once the hatch to the driven chamber was tightly sealed with the mouse inside the ABS, compressed air was allowed to accumulate in the driver end, until the pressure (150–160 psi) was great enough to rupture the membrane and the shock wave travelled down the ABS to where the animal was placed (about 2.9 m away from the membrane). After the blast wave was delivered, the mouse was removed from the mesh pocket and assessed for the occurrence of apnea. Sham-treated mice were placed in a clean cage in a supine position outside of the ABS, and the latency to recover the righting reflex was recorded for all mice. Once both ABS and sham animals regained consciousness, they were returned to their home cages and provided with acetaminophen (Tylenol) in their drinking water (1 mg/ml; ∼200 mg/kg b.w. for 24 h). Animals were weighed both immediately before and one day after ABS exposure.

### Behavioral Testing

**FIGURE 1:**
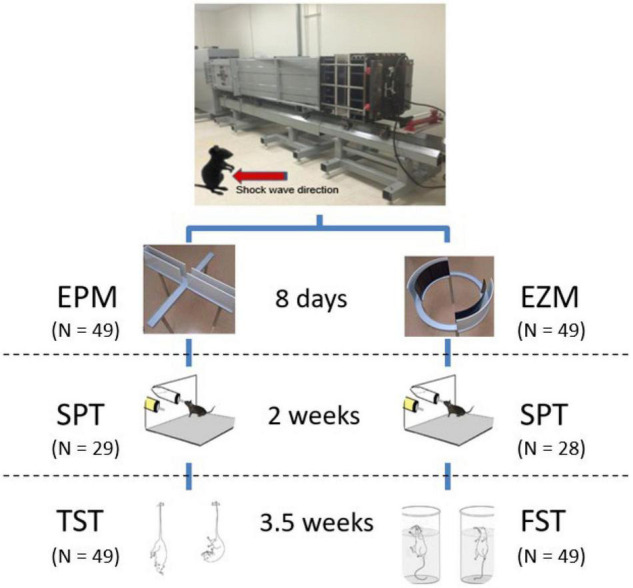
Advanced Blast Simulator and behavioral testing paradigms. A single shock wave (∼15 psi) was delivered with the mouse in an upright position with the ventral surface of the mouse exposed to the oncoming shock wave (see Vu et al., 2018 for a detailed description of the ABS). Eight days post-injury, animals underwent either EPM or EZM testing followed by the SPT 2 weeks post-injury or sham treatment, and lastly either TST or FST 3.5 weeks following ABS. Images reproduced from Abelaira et al. (2013), with permission from the Brazilian Journal of Psychiatry. Images reproduced from Brigman et al. (2010), Tucker and McCabe (2017).

Animals were randomly assigned to different behavioral task cohorts. The five cohorts consisted of *n* = 18–20 mice evenly distributed between males and females as well as injured and sham conditions (Figure 1). Following ABS as described above, each cohort underwent one of two testing paradigms: Elevated Plus Maze (EPM, 8 days post-injury) then Sucrose Preference Test (SPT, 2 weeks post-injury) and Tail Suspension Test (TST, 3.5 weeks post-injury), or Elevated Zero Maze (EZM, 8 days post-injury) then Sucrose Preference Test (SPT, 2 weeks post-injury) and Forced Swim Test (FST, 3.5 weeks post-injury).

### Elevated Plus Maze

The EPM (Stoelting, Wood Dale, IL, United States) was conducted 8 days following ABS to assess anxiety-like behavior (Pellow et al., 1985). The EPM is a cross-shaped platform with equal 35 cm length and 5 cm width arms raised 50 cm above the floor. Two opposite side arms are “open,” with 1 cm high edges and the remaining arms are “closed” with opaque, dark 16 cm high walls (Figure 1). Animals were allowed to acclimate to the room for 30 min before testing began. Overhead fluorescent lights illuminated the maze during testing with 1600 lux illuminance for the open arms and 200 lux for the closed arms. To start the test, individual mice were placed at the 5 cm center square region and allowed to explore the maze for 5 min. A ceiling camera and Any-Maze software (Stoelting) tracked animal movement throughout the test and were used to calculate the time spent in the open and closed arms, distance travelled, and number of entrances to the open arms during the session.

### Elevated Zero Maze

The Stoelting EZM (Shepherd et al., 1994) as previously described by Tucker et al. (2017) was performed 8 days post-injury to examine anxiety-like behavior. Briefly, the EZM is a 60 cm diameter ring platform raised 50 cm above the floor. The ring is divided into four equally sized areas with two opposite side “open” quadrants and two remaining “closed” quadrants (Figure 1). The open quadrants have 1 cm high edges and are exposed to 1600 lux light from overhead fluorescent lamps whereas the closed quadrants have 16 cm high dark, opaque walls and have a brightness of 200 lux. All quadrants have 5 cm width lanes. Animals spent 30 min acclimating to the room before starting the test. At the beginning of the test, mice were placed individually at an arbitrary boundary between an open and closed quadrant facing the closed quadrant. Animals were allowed to freely explore the maze for 5 min and a ceiling camera tracked the animal movement throughout the test. Any-Maze software was used to calculate the time spent in the open and closed quadrants, distance travelled, and the number of entrances to the open quadrants during the test.

### Sucrose Preference Test

The SPT was administered 2 weeks following injury as a measure of anhedonia. Due to laboratory spatial and time constraints, housed mice cages were randomly selected so that ∼57% of the mice were tested. Cages of mice not selected for testing remained group housed. Individually housed mice were evaluated over the course of 5 days. Mice were offered two 20 mL bottles of 1% sucrose diluted with water placed about 7.5 cm apart at equal heights in their home cages for the first 2 days of testing to acclimate them to the sweet taste and to the new bottles. The amount of sucrose consumed was measured by weighing each bottle daily. On the third day, one bottle was replaced with filtered tap water, so that each mouse was given a choice of drinking solutions of sucrose or tap water. On the fourth day, the positions of the bottles were switched to control for potential side bias. SPT bottles were again weighed and the sucrose preference ratio was calculated for the final 48 h of testing with the following equation: sucrose preference ratio =


c⁢o⁢n⁢s⁢u⁢m⁢e⁢d⁢s⁢u⁢c⁢r⁢o⁢s⁢ec⁢o⁢n⁢s⁢u⁢m⁢e⁢d⁢w⁢a⁢t⁢e⁢r+c⁢o⁢n⁢s⁢u⁢m⁢e⁢d⁢s⁢u⁢c⁢r⁢o⁢s⁢e⁢x⁢ 100

### Tail Suspension Test

The TST to measure depressive-like behavior was performed 3.5 weeks after ABS. As previously described by Can et al. (2012), mice were suspended from their tails from laboratory benches with tape (12 mm wide, 24 cm long). The tape was adhered about 1 cm from the tip of the tail and a 4 cm length hollow polycarbonate tube (1.3 cm inner diameter, McMaster-Carr, Santa Fe Springs, CA #8585K41) was placed around the base of the tail to prevent tail-climbing during the test. Padding was placed below the mice in case of falls and mice were monitored throughout the 6 min test. A standard video camera was used to record the sessions and videos were uploaded into Any-maze. The time spent immobile (defined as animals with minimal movement) during the last 5 min of the session to account for initial test acclimation was later scored using Any-Maze with manual key presses by an investigator blinded to injury condition where a computer key was held down for the duration of animal immobility.

### Forced Swim Test

The Porsolt FST (Porsolt et al., 1977) to study learned helplessness was conducted 3.5 weeks post-injury. As described by Tucker et al. (2017), FST chambers (Stoelting) were clear 42 cm in height and 19 cm diameter Plexiglas cylinders. The chambers were filled to a depth of about 25 cm with water at 25°C. Mice were placed into the cylinders for 6 min and allowed to swim or float. Mice were closely monitored from a separate room. Once the test concluded, mice were gently dried with paper towels and placed in a clean cage under a heat lamp to dry. FST cylinders were rinsed and replaced with fresh water for each animal. A standard video camera was used to record sessions and videos were imported into Any-Maze with key presses to measure immobility (defined as animals floating on the water surface with minimal movements). The first minute of the test was not scored to account for initial acclimation to the FST, so an investigator blinded to injury condition only scored the last 5 min of the test.

### Immunohistochemistry

A separate cohort of animals, which did not undergo behavioral testing, were exposed to a single ABS shock wave (about 15 psi) and examined for the presence of Evans blue as a marker of blood-brain barrier disruption. An animal restrainer was used to intravenously administer Evans blue (2% diluted in buffer, 0.1 mL per animal) via the tail veil immediately prior to ABS exposure. The mice were euthanized 4 h (*n* = 25, *n* = 6–7 per injury and sex condition) or 24 h (*n* = 15, with Evans blue injection after ABS) post-injury and tissue was collected for c-Fos immunohistochemistry from only the 4 h group. Briefly, mice were anesthetized with a mixture of ketamine and xylazine and then transcardially perfused with cold phosphate buffer solution (0.1 M) and 4% paraformaldehyde in phosphate buffer. Brains were dissected and further fixed in paraformaldehyde for an additional 24 h. They were then transferred to 20% sucrose solution in phosphate buffer for 72 h before freezing the tissue and storage (−80°C) until sectioning. A Leica microtome was used to cut 30 μm thick coronal sections. The tissue was initially washed in tris-buffered saline with 0.05% triton (TBS-T). Sections were then processed with 0.3% hydrogen peroxide for 30 min and afterwards washed with TBS-T again before blocking buffer (TBS-T with 0.20% triton, goat serum, and 10% bovine serum albumin; BSA) incubation for 1 h at room temperature. C-Fos (1:1000; Millipore Sigma Cat: ABE457, Lot: 3585299) antibody was applied to the sections before storage at 4°C overnight. The next day, sections were washed with TBS-T and secondary antibody goat-anti-rabbit IgG (1:1000 Jackson Immunoresearch Cat: 111-065-003, Lot: 117316) was applied in blocking buffer (TBS-T with 0.05% triton, goat serum, and 10% BSA) for 1 h at room temperature. Sections were again washed with TBS-T before incubation in ABC solution (Vectastain ABC HRP Kit, PK-4000, Vector Laboratories) for 45 min at room temperature. The tissue was washed a final time with TBS-T prior to DAB development with the DAB Substrate Kit, Peroxidase (HRP), with Nickel, 3,3′-diaminobenzidine (SK-4100, Vector Laboratories) for 1 min. The free-floating sections were mounted onto glass slides and left to dry overnight. Lastly, sections were dehydrated in ethanol gradients (75–100%), cleared in xylene, and cover slipped with Permount mounting media the next day for analysis. Positive, activated neural tissue from restrained ABS-exposed mice, and negative, tissue processed without primary antibody, controls were included in all immunohistochemistry procedures.

Three regions of interest (ROIs), bilateral cerebral cortex, bilateral amygdala, and the PVT, were analyzed for c-Fos staining. Six mice per group were selected for Carl Zeiss El-Einsatz model #451485 light microscope imaging. Images for the PVT were taken at 100× magnification, the cerebral cortex at 50×, and the amygdala was captured at 25× magnification. To quantify c-Fos in each ROI, the threshold and particle analysis functions on Image J software (NIH) were used for cell counts on black/white c-Fos images. The particle count was employed as an estimate for the number of c-Fos positive cells. The values were averaged for three or more sections per animal. Cresyl violet (Chroma-Gesellschaft Schmid GmbH & Co 1 A 396) and H&E (haematoxylin Sigma-Aldrich Cat: GHS132-1L, Lot: SLCH6216 and eosin Sigma-Aldrich Cat: HT110332-1L, Lot: SLCJ2544) staining were performed to indicate anatomical consistency for the ROIs and for microbleed analysis, respectively.

### Statistics

GraphPad Prism version 8.42 (GraphPad Software, San Diego, CA, United States) and SPSS version 27.0.1.0 (IBM, Armonk, NY, United States) were used for statistical analysis and figure generation. Body weights were measured on the day of injury and 24 h later. Since there was a noticeable difference in baseline (before injury) body weights, a two-way Injury × Sex analysis of variance (ANOVA) was performed for the mice as a percentage of body weight loss after sham or injury treatment. Mann-Whitney U tests were performed to analyze righting time data using within sex comparisons and between sex comparisons for male ABS and female ABS groups. Photographs of the brains from the cohort of mice that were euthanized after Evans blue infusion and ABS exposure were visually ranked for degree of staining. The rankings for Evans blue staining intensity were evaluated with respect to righting reflex to determine if there was an association between staining and the duration of the righting reflex. Due to smaller samples sizes and score ties, the SPSS program to determine Kendall’s tau (τ) was employed and the SPSS bootstrap procedure for estimation of the 95% confidence interval for the correlation coefficient, based upon 1000 bootstrapped samples, was performed.

Two-way ANOVAs (injury × sex) were performed for all behavioral tasks (EPM, EZM, SPT, FST, and TST). Behavior graphs depict the scores for individual animals and the embedded horizontal bar shows the arithmetic means for each group. Histology for c-Fos was analyzed using two-factor (injury × sex) ANOVAs. *p* < 0.05 was considered significant.

## Results

### Shock Wave Characteristics, and Mouse Body Weight, Righting Times, Apnea, Morbidity

**FIGURE 2:**
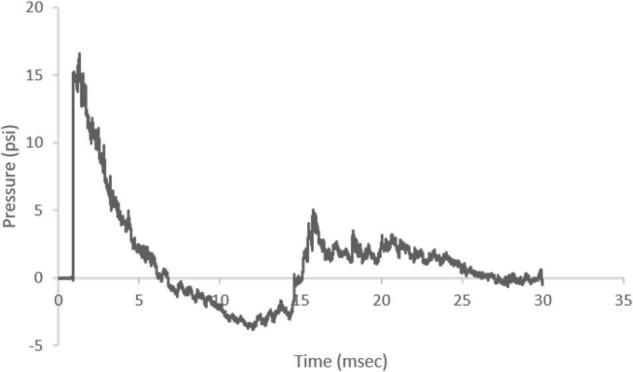
Representative Friedlander-like ABS waveform. The ABS device produced a waveform with an average of 15.56 psi peak incident pressure and 469.74 m/s shock wave velocity. The shock wave positive phase duration was 5.62 ms and negative phase duration was 8.40 ms.

The ABS produced a characteristic Friedlander-like curve with a consistent peak pressure across the study (mean 15.56 psi, coefficient of variation = 4.5%) (Figure 2). The shock wave velocity was approximately 469.73 m/s and the positive and negative phases were 5.62 and 8.40 ms, respectively, with an average impulse (pressure × time) of 0.0358 psi × s.

**FIGURE 3:**
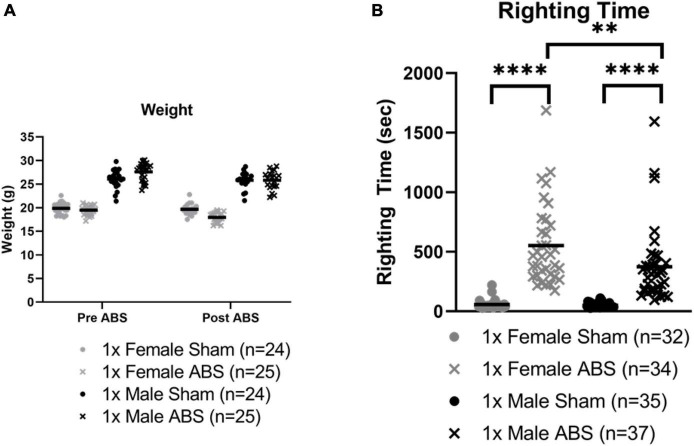
Weight and righting time changes after bTBI. **(A)** Animal weight before and one day after ABS exposure for the behavior cohort of mice. Due to differences in baseline body weights, the percentage change in body weight for each mouse was computed for pre- and post-injury sham or ABS treatment (data not shown; see text). ANOVA indicated there was a significant greater difference in females compared to males overall and a significantly greater percentage of body weight loss in ABS animals, but no sex differences due to injury. **(B)** Righting times of all cohorts, behavior and Evans blue, immediately after a single blast. The Mann Whitney U test (*U* = 3, *p* < 0.0001) demonstrated that the duration of the righting reflex was longer for shock wave exposed females compared to female sham mice. Male injured mice also demonstrated a longer righting reflex compared to sham counterparts (*U* = 1.5, *p* < 0.0001). A comparison of righting reflex duration in injured mice indicated the responses after shock wave exposure in males and females were significantly different (*U* = 370.5, *p* = 0.0026) with longer righting times for female mice. Bars indicate means. *p ≤ 0.05, **p ≤ 0.01, ****p ≤ 0.0001. No significant correlations were found between righting reflex and loss of body weight (τ = 0.000, *p* = 1.000) or percentage loss of body weight (τ = 0.032, *p* = 0.846) for females, or righting reflex and loss of body weight (τ = –0.167, *p* = 0.312) or percentage loss of body weight (τ = –0.105, *p* = 0.516) for males.

Figure 3A summarizes the body weights of individual female and male mice before and after sham or ABS exposure. Since the baseline (preinjury) body weights for the mice in the ABS and sham groups were not equivalent, an Injury × Sex ANOVA was computed using the percentage change in body weights after ABS injury or sham treatment compared to pre-injury body weights. The average percent change in body weight was −2.23 and 0.574% for the female and male mice, respectively, on the day after sham treatment, while the females lost −8.22 and the males −5.79% of their preinjury body weight after ABS exposure. The ANOVA indicated there was a significant overall main effect between the females and males in percentage of body weight loss before to after sham or ABS treatment (−2.619% change, *F*_1,76_ = 24.214, *p* < 0.001) and a difference in the percentage of body weight loss after ABS injury compared to sham treatment (−7.002%, *F*_1,76_ = 134.584, *p* < 0.001), but no interaction effect to suggest the injuries had a different effect on female and male mice (*F*_1,76_ = 0.123, *p* = 0.727).

Behavior and Evans blue-treated cohorts were combined for righting reflex analysis. Mann-Whitney U analyses showed injured animals required a longer time to regain consciousness compared to sham mice. Female shock wave exposed mice (462.5 s median) had increased righting times compared to female sham mice (46.0 s median; *U* = 3, *p* < 0.0001) and male shock wave exposed mice (316.0 s median) displayed longer righting reflexes than male sham animals (43.0 s median; *U* = 1.5, *p* < 0.0001). A third Mann-Whitney test showed injured male and female mice were significantly different, with male animals exhibiting overall shorter reflex durations than female mice (*U* = 370.5, *p* = 0.0026, Figure 3B). Five cases of apnea, ranging from about five to thirty seconds, were observed in two male and three female injured animals. One case with about 5 s of apnea corresponded to the longest female ABS righting time. Six animals died following ABS. Five female mice died immediately after injury and one male mouse died 4 days after ABS exposure.

### Anxiety-Like Behavior

**FIGURE 4:**
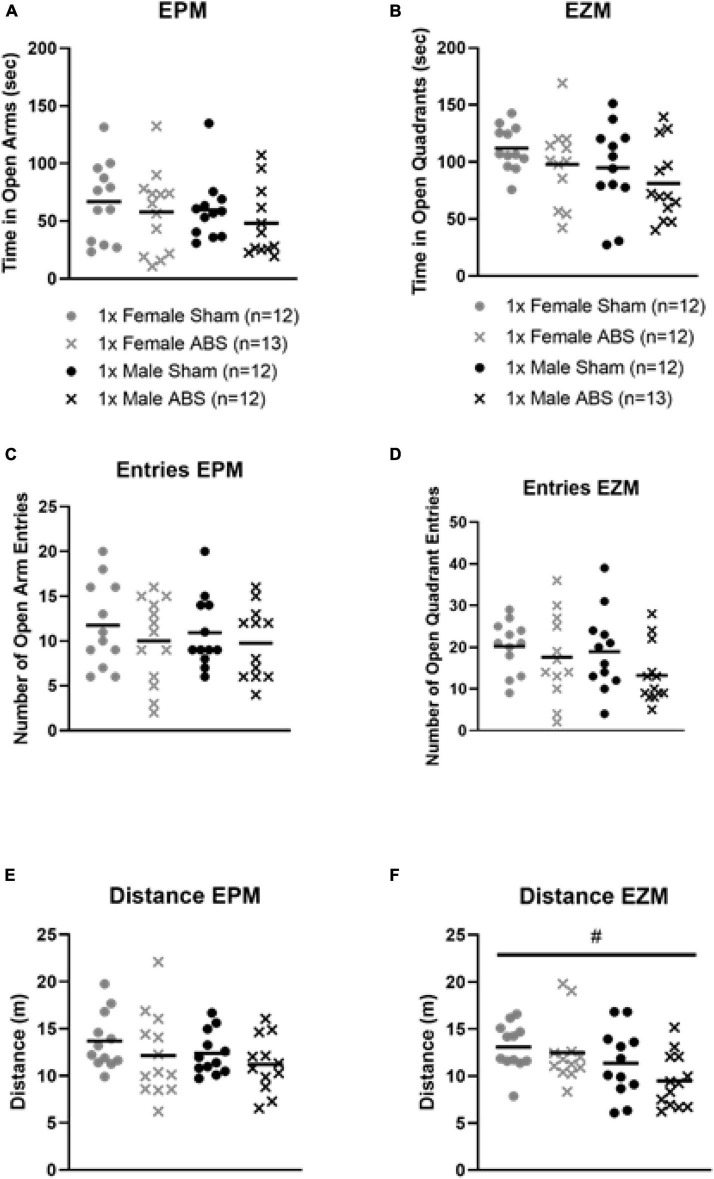
No changes in anxiety-like behaviors on the EPM of EZM after injury, but a sex difference was displayed for EZM distance travelled. The results from the **(A)** EPM and **(B)** EZM testing show no difference in terms of time spent in the open areas for injury or sex. The number of open region entries also did not significantly differ for **(C)** EPM or **(D)** EZM. ABS group and sham animals for each behavioral task. **(E)** The total distance travelled was not statistically different between injured (ABS) or sham mice for EPM. **(F)** Results from the EZM testing indicated there was a main effect of sex where females travelled significantly farther than male mice (*p* = 0.0112). Bars indicate means. # indicates main effect of sex *p* ≤ 0.05.

For the EPM and EZM, injured mice overall spent less time in the open regions, but the two-way ANOVAs were not statistically significant (Figures 4A,B). No statistical differences were detected for either EPM or EZM test in terms of the amount of time spent in or the number of entries into the open areas (Figures 4C,D). No difference in the total distance travelled during EPM testing was evident, but for the EZM there was a main effect of sex in which female animals travelled a greater distance compared to male mice (*F*_1,45_ = 6.999, *p* = 0.0112, Figures 4E,F).

### Depressive-Like Behavior

**FIGURE 5:**
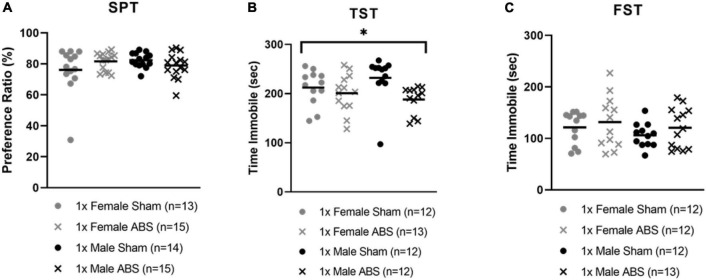
Decreased time immobile on the TST following bTBI. **(A)** SPT did not show any significant difference in sucrose consumption as an effect of shock wave injury or sham treatment. **(B)** TST displayed a significant effect of injury with ABS mice spending less time immobile compared to sham mice. **(C)** The time immobile on the FST was not significant. Bars indicate means. *p ≤ 0.05 for main effect for the difference between uninjured and ABS exposed mice.

The two-way ANOVA for the SPT data did not detect statistical differences between the sham and injured or male and female mice for sucrose intake. However, the two-way ANOVA for the TST data showed a main effect of injury (*F*_1,45_ = 6.763, *p* = 0.0125) with shock wave exposed animals spending less time immobile than sham-treated mice. No sex differences were reported for the TST. The FST two-way ANOVA indicated no group differences for immobility (Figure 5).

### Behavioral Testing Sequence Effects

*Post hoc* analyses to determine whether or not prior behavioral tests affected later testing performance was assessed. Specifically, we evaluated whether or not the EZM or EPM may have affected performance on the SPT, and if subgroups of mice that were or were not evaluated on the SPT may have affected performance on the subsequent TST and FST. The performance ratio measures in mice that previously received testing on the EPM and EZM were compared using a three-factor (Injury × Sex × EPM/EZM Testing). ANOVA indicated there was no significant main effect of EPM vs. EZM on the subsequent SPT (*F*_1,49_ = 1.131, *p* = 0.293). Likewise, a three-factor ANOVA (Injury × Sex × SPT Testing/No SPT Testing) indicated there was no difference on either the TST or the FST as a function of mice having been tested on the SPT (and single housing) compared to mice that had not been used on this test (and remained in group housing). The main effect for Injury on the TST was significant, as expected (*F*_1,41_ = 8.206, *p* = 0.007), indicating the time immobile on the TST was less in injured mice compared to the sham animals. For the FST, there were no significant differences between groups on any factors.

### Histology

#### Evans Blue Staining

**FIGURE 6:**
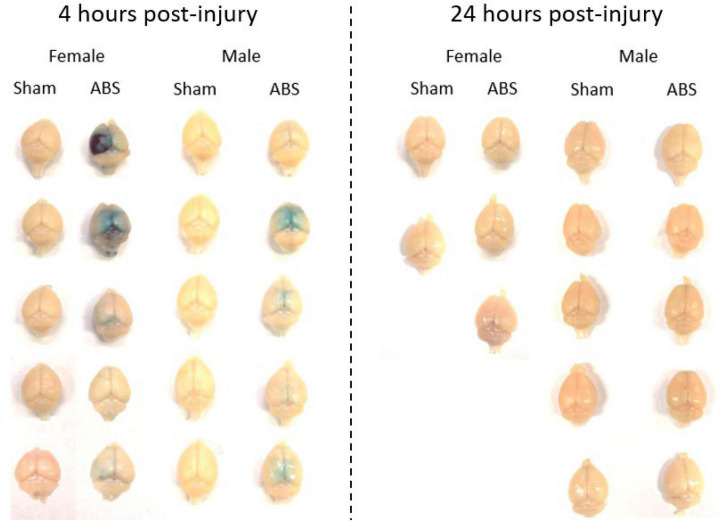
Evans blue was evident 4 but not 24 h after shock wave exposure. Evans blue staining was present on the brain parenchyma of ABS-exposed male and female mice 4 h after injury, but was not apparent on the brains of sham animals. One day after shock wave exposure, Evans blue was not observed on the brain parenchyma of either ABS-injured male and female or sham animals.

**FIGURE 7:**
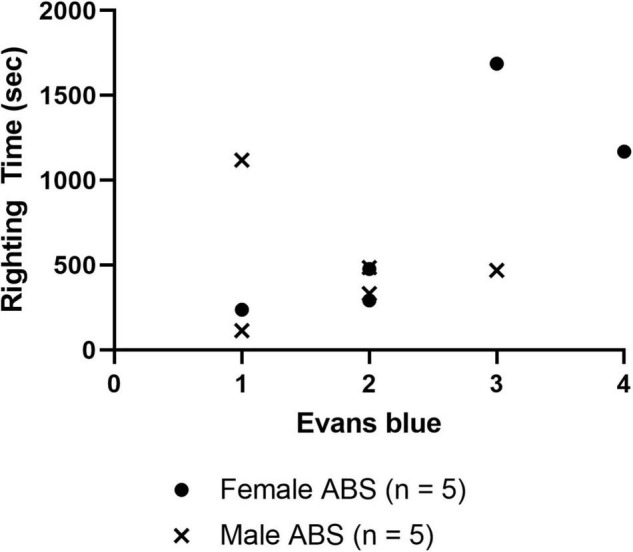
Correlation of righting reflex time and Evans blue staining 4 h post-injury. The scatterplot displays the righting time of injured male and female mice 4 h after shock wave exposure with respect to the amount of Evans blue staining intensity ranked 1 (least) –4 (most), and Kendall tau analysis indicated a significant correlation between stain intensity and the duration of the righting reflex (τ = 0.508, *p* = –0.030). No correlations (data not shown) were detected between Evans blue ranking and peak ABS pressure (τ = –0.025, *p* = 0.925), Evans blue and body weight (τ = 0.090, *p* = 0.719), righting time and peak pressure (τ = 0.244, *p* = 0.325).

Evans blue staining was evaluated in a separate cohort of sham and injured animals 4 h post-ABS. No staining appeared on the brain parenchyma of sham animals (Figure 6). Of note was the observed variability in Evans blue deposition in the cerebral cortex following injury, and there was a trend (albeit with a small sample size) for the appearance of a more intense uptake in some females. There appeared to be one case of subdural hemorrhage isolated in the left hemisphere of an injured female (top photograph for the Female ABS mice in Figure 6) and general staining near the superior sagittal sinus in several animals. A smaller cohort of mice was analyzed for the presence of Evans blue 24 h after shock wave exposure, but no staining was evident in either the injured or sham animals (Figure 6). Kendall’s tau was computed to determine whether or not there was an association between the intensity of Evans blue staining 4 h after ABS injury and the duration of the righting reflex. There was a significant association, where longer righting reflex duration was associated with more intense Evans blue staining (τ = 0.580, 95% confidence interval = 0.029–0.924, *p* = 0.030). A summary of the measures is presented in Figure 7.

#### H&E and Cresyl Violet

Qualitative analysis of H&E did not reveal any microbleeds in any brain regions in the samples from ABS or sham animals (data not shown). Adjacent sections immunolabeled for c-Fos were stained with Cresyl Violet to confirm consistent anatomical location of the amygdala (data not shown).

#### c-Fos

**FIGURE 8:**
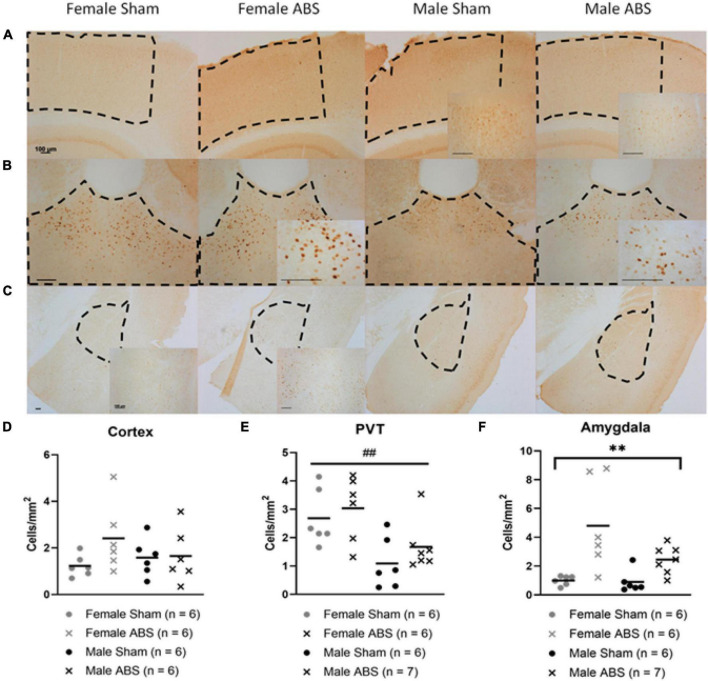
c-Fos differences by sex and injury in the PVT and amygdala, respectively. Representative images of the **(A)** cerebral cortex, **(B)** PVT, and **(C)** amygdala (basolateral and central nucleus) with regions of interest outlined in dashed lines and magnified inserts of areas within the measured regions. All scale bars indicate 100 μm. **(D)** The cerebral cortex did not show any significant difference between injury or sex. **(E)** The PVT displayed an overall effect of sex in which females had more c-Fos positive cells compared to males. **(F)** There was a main effect of injury in the amygdala, where injured mice had greater c-Fos cell density than sham mice. Bars indicate means. ## indicates main effect of sex *p* ≤ 0.001, ** indicates main effect of injury *p* ≤ 0.001.

Data are presented collapsed across anatomical sides. No significant differences were observed in the cerebral cortex (Figures 8A,D). There was a main effect of sex for staining in the PVT (*F*_1,14.497_ = 15.781, *p* = 0.001) with females exhibiting more c-Fos staining compared to male mice (Figures 8B,E). There was a main effect of injury in the amygdala (*F*_1,44.567_ = 16.036 *p* = 0.001) with ABS-injured mice expressing more c-Fos positive cells compared to sham mice (Figures 8C,F).

## Discussion

### Physiological Changes After Blast Traumatic Brain Injury

The current study found that male and female mice experienced longer righting reflex times after shock wave exposure compared to sham animals, and female ABS-exposed mice expressed longer periods of unconsciousness than male injured mice. The sex differences observed for righting times may be correlated with variations in body weight. As expected, males were larger than females, which may affect injury-related biomechanics during the shock wave exposure, causing the smaller female mice to move more during the blast event. The decrease in body weight following shock wave, as well as the increase in righting reflex for animals in the injured condition, indicate that the shock wave produced physiological effects. The loss in body weight suggested that, acutely, injured animals were physically unable or uninterested in eating following ABS. The observed weight loss and longer righting reflexes after ABS are consistent with a recent ABS study in the laboratory with identical conditions (Nonaka et al., 2021), as well as other reports (Schindler et al., 2020). Previous experiments demonstrated only transient changes in water and food consumption post-blast with vertical orientation exposure, or no differences in male and female body weight after ABS when animals were placed in a prone position (Russell et al., 2018b; Vu et al., 2018). The sex differences in weight and righting time immediately following bTBI suggest that female mice sustained greater damage initially compared to male mice.

### Effects of Blast Traumatic Brain Injury on the Blood-Brain Barrier

Behavioral and neuropathological changes have been reported in blast studies in which the animal is placed in a vertical orientation (Koliatsos et al., 2011; Vu et al., 2018; Nonaka et al., 2021) and in a prone position (Bailey et al., 2016; Sawyer et al., 2016; Russell et al., 2018a; Arun et al., 2020). Body orientation may be particularly relevant to cases of tertiary blast effects. The BBB, for example, is an essential element in brain homeostasis and is one of the first sites to be altered following bTBI, and is a structure particularly vulnerable to impact compression (Gama Sosa et al., 2014; Huber et al., 2016). Acute cerebral vascular impairment and BBB protein dysregulation have been reported after single and multiple ABS exposure, respectively (Heyburn et al., 2019, 2021; Rodriguez et al., 2019), and our data support these findings with acute BBB permeability to Evans blue 4 h following ABS. Finite element modeling and shock tube experiments demonstrate that the prone orientation produces a lesser degree of damage compared to other body orientations (Hubbard et al., 2014; Heyburn et al., 2019; Unnikrishnan et al., 2021). In this study the vertical orientation was employed and Evans blue staining provided evidence of BBB disruption, but it was evident only on the dorsal surface of the brain. This suggests that the fixed vertical animal positioning enabled tertiary, acceleration artifacts from the shock wave-associated blast wind that pushed the mouse backwards, compressing the top of the head into the mesh holder. Interestingly, (Unnikrishnan et al., 2021) used almost identical peak pressure (100 kPa or 14.5 psi) modeling of vertical orientation inside of the ABS, and reported higher pressure on the ventral brain than the dorsal side.

Assessment of the degree of Evans blue staining on the dorsal surface of the neuraxis, 4 h after ABS injury, was associated with the duration of the righting reflex, suggesting longer righting reflex duration was reflected in greater severity of BBB compromise (Figure 7). However, although Evans blue staining was present in some injured animals 4 h after bTBI, H&E staining did not indicate the presence of microbleeds and no c-Fos changes were observed in the cerebral cortex, suggesting that the extent of the damage was limited to albumin extravasation to the superficial surface, but not deeper cortical layers (Weng et al., 2011). The damage, however, might be transient since only subtle changes in behavior were observed at later time points (Yang et al., 2016). Another study demonstrated the extravasation of dyes smaller than 70 kDa with barrier integrity restoration one day post-blast (Hue et al., 2015), which is consistent with the observed absence of Evans blue 24 h following shock wave exposure in the current study, indicating that the BBB may be repaired in rodents one day after bTBI. Evans blue has been used historically, but caution must be used with extravasation interpretation and its quantification continues to present challenges (Saunders et al., 2015). Taken together, many potential artifacts are present in preclinical bTBI designs and additional complications with Evans blue quantification, including observed variability in stain intensity, hinder definitive conclusions from being made regarding BBB disruption in this study.

#### Acute Changes in c-Fos Activation in the Amygdala and Paraventricular Nucleus of the Thalamus Following Blast Traumatic Brain Injury

In addition to BBB disruption, there were increases in c-Fos activation in the amygdala (males and females) following TBI and overall in the PVT (females only). The c-Fos activation reported in this study supports previous investigations of behavioral changes after blast injury through limbic activation. In previous studies, increases in c-Fos appeared in the central and basolateral amygdala and subsequent increases occurred in anxiety-like behavior and fear circuitry (Rowson and Pleil, 2021; Ou et al., 2022). Reports of changes in cytostructure after bTBI also may shed light on possible mechanisms of hyperactivity in the amygdala with increased dendritic branching and spine density after injury (Ratliff et al., 2019, 2020). The sex differences in c-Fos seen in the PVT may correspond to the higher hypothalamic-pituitary-adrenal axis activation in females and maladaptive behaviors in response to stress (Ishii et al., 2018; Rowson and Pleil, 2021). The acute changes in neuronal activation observed primarily in the amygdala after ABS and overall c-Fos sex differences in the PVT were consistent with some of the behavioral alterations seen at later time points.

### Effects of Blast Exposure on Depressive- and Anxiety-Like Behaviors

The mild and transient nature of the bTBI as assessed histologically was associated with only subtle changes in behavior. Previous bTBI studies described increased anxiety-like behavior in the EZM and EPM in injured rats and mice (Elder et al., 2012; Xie et al., 2013; Awwad et al., 2015; Russell et al., 2018a). However, the present study did not find significant differences for time in the open region, nor for the number of entrances into the open zones for EPM and EZM. Some of the differences in reported anxiety-like behavior could be due to the type of shock tube used. One group reporting anxiety-like behaviors following bTBI used a compact, focal blast tube with animal head-only exposure (Awwad et al., 2015), whereas another group used a full body shock tube, but with multiple blast injuries (Elder et al., 2012). A different design of repetitive, automated blast exposures was implemented in an air blast chamber with anxiety-like behavior performed 5 min after injuries (Xie et al., 2013). Russell et al. (2018a) employed an injury paradigm that closely resembled the current study, but animals were placed in a prone instead of vertical orientation. These differences in animal body orientation and blast tube design alter injury biomechanics and therefore functional outcomes (Needham et al., 2015; Unnikrishnan et al., 2021).

While there was no change in the total distance travelled in the EPM, females travelled farther than males in the EZM. Hyperactivity has been observed in females during open field tests following controlled cortical impact (Tucker et al., 2016, 2017). Likewise, a recent study with repetitive concussive injuries directed at the frontal region likewise observed increased distance travelled in the open field test, and that female mice travelled farther in an acute test 4 days after injury, but not at later time points (Vu et al., 2021). The higher levels of activity, particularly in females as observed in the EPM and in open field in other studies, could be related to activational effects of estrogen (Morgan and Pfaff, 2002; Ogawa et al., 2003), but further investigation is needed to understand the mechanism and duration of this increased activity in female mice after blast.

Behavioral tasks measuring depressive-like behaviors showed varying trends. No sex differences were found, but the injured group had reduced immobility in the TST compared to sham-treated controls, an unexpected finding. Reduced immobility after blast exposure may indicate injury-induced agitation or a panic-like reaction and has been reported after other concussive TBI models (Anyan and Amir, 2018; Tucker et al., 2019). Interestingly, a study of CRF neurons after bTBI and restraint-induced stress found sex differences with females showing increased amygdala CRF2 gene expression and hippocampal decreased expression, whereas males displayed the opposite (Russell et al., 2018a). Increased neuronal activation in the amygdala of injured females was consistent with the current study. Hyperarousal is an element of PTSD and as discussed previously, blast is able to induce PTSD-like traits where greater activity levels during the TST in injured animals may correspond to the hyperactivity observed in female mice in the EZM (Elder et al., 2012; Perez-Garcia et al., 2019). Activity level, however, has been criticized as an inaccurate measurement of anxiety (Lister, 1990; Tucker and McCabe, 2021). The TST and FST are arguably models of acute stress, which could be interpreted as changes in anxiety rather than depression (Lister, 1990; Nestler and Hyman, 2010; Tucker et al., 2017; Anyan and Amir, 2018). Further study is needed to define the relationship between bTBI and subsequent hyperactivity.

### Study Limitations and Future Prospects

Results from behavioral tests can depend on the animal strain and sex (McCabe and Tucker, 2020). Overall, female rodents exhibit more activity compared to males during behavioral tasks and this inherent difference can make data interpretation challenging (Tucker et al., 2016, 2017). Females may also have subtle neuroprotective effects associated with estrogen and progesterone, but estrous cycle stage does not appear to have a predominant effect on behavioral outcome after TBI (Wagner et al., 2004). Likewise, housing conditions with respect to sex differences in behavior should also be considered. Animals in the current study were singly housed for the SPT and remained individually housed for subsequent testing with the TST and FST. In this study, single housing did not affect depressive-like behavior. Increased corticosterone and anxiety-like behavior have been reported in female rodents that are singly housed, whereas individually housed males display less anxiety-like behavior (Brown and Grunberg, 1995; Palanza et al., 2001). A potential confound of this study’s histological data included additional animal restraint during intravenous Evans blue administration, but injured and sham-treated mice underwent the same injection procedure. Previous literature demonstrated that blast alone without a stressor could increase contextual fear conditioning (Elder et al., 2012; Perez-Garcia et al., 2019; Perez Garcia et al., 2021). TST and FST alone can cause changes in c-Fos activation (Yanagida et al., 2016; Hiraoka et al., 2017), so separate cohorts of animals were included for behavioral testing and pathology in this study, and no behavioral stress confounds occurred for c-Fos staining (Cullinan et al., 1995; Yanagida et al., 2016; Hiraoka et al., 2017). Since c-Fos activation has been reported in other areas after bTBI, such as the paraventricular nucleus of the hypothalamus and hippocampus, additional brain regions should be investigated (Du et al., 2013; Russell et al., 2018b; Ou et al., 2022) and given reports of different PVT cellular divisions, specific PVT circuitry should be identified (Kirouac, 2015; Gao et al., 2020). Future directions could also include investigation of multiple blast exposures and bTBI with chronic stress as more clinically relevant designs for military populations (Owens et al., 2008; Kontos et al., 2013). The evaluation of later time points after blast for behavioral tasks may indicate delayed or biphasic behavioral changes, such as slowed onset or immediate deficits followed by a recovery and then prolonged alterations (Stemper et al., 2016; Arun et al., 2020; Perez Garcia et al., 2021). This study further highlights the importance of investigating potential sex differences, and indicates overall variance in limbic activation and functional outcome between male and female rodents following bTBI exposure.

## Data Availability Statement

The raw data supporting the conclusions of this article will be made available by the authors, without undue reservation.

## Ethics Statement

The animal study was reviewed and all procedures were approved by the Uniformed Services University of the Health Sciences Institutional Animal Care and Use Committee.

## Author Contributions

EM, LT, and JM contributed to the experiment design and development and edited the manuscript. PV assisted with ABS training. EM, AF, and YK performed the ABS procedures. EM and LT conducted the behavioral testing. AF assisted with tail injection training and performed tail injections. EM and JL performed the histology. EM and JM performed the statistical analyses and wrote the manuscript and finalized the manuscript. All authors contributed to the article and approved the submitted version.

## Author Disclaimer

The opinions expressed herein are those of the authors and are not necessarily representative of those of the Uniformed Services University of the Health Sciences (USUHS), the Department of Defense (DOD), the NIH or any other U.S. government agency, or the Henry M. Jackson Foundation, Inc. (HJF). The opinions, interpretations, conclusions and recommendations are those of the authors and are not necessarily endorsed by the U.S. Army, Department of Defense, the U.S. Government or the Uniformed Services University of the Health In review Sciences. The use of trade names does not constitute an official endorsement or approval of the use of reagents or commercial hardware or software. This document may not be cited for purposes of advertisement.

## Conflict of Interest

The authors declare that the research was conducted in the absence of any commercial or financial relationships that could be construed as a potential conflict of interest.

## Publisher’s Note

All claims expressed in this article are solely those of the authors and do not necessarily represent those of their affiliated organizations, or those of the publisher, the editors and the reviewers. Any product that may be evaluated in this article, or claim that may be made by its manufacturer, is not guaranteed or endorsed by the publisher.

## References

[B1] Abelaira H. M. Reus G. Z. Quevedo J. (2013). Animal models as tools to study the pathophysiology of depression. *Braz. J. Psychiatry* 35(Suppl. 2), S112–S120. 10.1590/1516-4446-2013-1098 24271223

[B2] Anyan J. Amir S. (2018). Too depressed to swim or too afraid to stop? A reinterpretation of the forced swim test as a measure of anxiety-like behavior. *Neuropsychopharmacology* 43 931–933. 10.1038/npp.2017.260 29210364 PMC5854810

[B3] Arun P. Wilder D. M. Eken O. Urioste R. Batuure A. Sajja S. (2020). Long-term effects of blast exposure: a functional study in rats using an advanced blast simulator. *J. Neurotrauma* 37 647–655. 10.1089/neu.2019.6591 31595810

[B4] Awwad H. O. Gonzalez L. P. Tompkins P. Lerner M. Brackett D. J. Awasthi V. (2015). Blast overpressure waves induce transient anxiety and regional changes in cerebral glucose metabolism and delayed hyperarousal in rats. *Front. Neurol.* 6:132. 10.3389/fneur.2015.00132 26136722 PMC4470265

[B5] Azevedo H. Ferreira M. Mascarello A. Osten P. Guimarães C. R. W. (2020). Brain-wide mapping of c-fos expression in the single prolonged stress model and the effects of pretreatment with ACH-000029 or prazosin. *Neurobiol. Stress* 13:100226. 10.1016/j.ynstr.2020.100226 32478146 PMC7251424

[B6] Bailey Z. S. Grinter M. B. Vandevord P. J. (2016). Astrocyte reactivity following blast exposure involves aberrant histone acetylation. *Front. Mol. Neurosci.* 9:64. 10.3389/fnmol.2016.00064 27551260 PMC4976110

[B7] Barson J. R. Mack N. R. Gao W.-J. (2020). The paraventricular nucleus of the thalamus is an important node in the emotional processing network. *Front. Behav. Neurosci.* 14:598469. 10.3389/fnbeh.2020.598469 33192373 PMC7658442

[B8] Blaze J. Choi I. Wang Z. Umali M. Mendelev N. Tschiffely A. E. (2020). Blast-related mild TBI alters anxiety-like behavior and transcriptional signatures in the rat amygdala. *Front. Behav. Neurosci.* 14:160. 10.3389/fnbeh.2020.00160 33192359 PMC7604767

[B9] Brigman J. L. Graybeal C. Holmes A. (2010). Predictably irrational: assaying cognitive inflexibility in mouse models of schizophrenia. *Front. Neurosci.* 4:13. 10.3389/neuro.01.013.2010 20859447 PMC2938983

[B10] Brown K. J. Grunberg N. E. (1995). Effects of housing on male and female rats: crowding stresses males but calms females. *Physiol. Behav.* 58 1085–1089. 10.1016/0031-9384(95)02043-8 8623006

[B11] Can A. Dao D. T. Terrillion C. E. Piantadosi S. C. Bhat S. Gould T. D. (2012). The tail suspension test. *J. Vis. Exp.* 59:e3769. 10.3791/3769 PMC3353516 22315011

[B12] Cernak I. Wang Z. Jiang J. Bian X. Savic J. (2001). Ultrastructural and functional characteristics of blast injury-induced neurotrauma. *J. Trauma* 50 695–706. 10.1097/00005373-200104000-00017 11303167

[B13] Chaudhuri A. (1997). Neural activity mapping with inducible transcription factors. *Neuroreport* 8 v–ix. 9427298

[B14] Cullinan W. E. Herman J. P. Battaglia D. F. Akil H. Watson S. (1995). Pattern and time course of immediate early gene expression in rat brain following acute stress. *Neuroscience* 64 477–505. 10.1016/0306-4522(94)00355-9 7700534

[B15] Curran T. Morgan J. I. (1995). Fos: an immediate-early transcription factor in neurons. *J. Neurobiol.* 26 403–412. 10.1002/neu.480260312 7775973

[B16] Du X. Ewert D. L. Cheng W. West M. B. Lu J. Li W. (2013). Effects of antioxidant treatment on blast-induced brain injury. *PLoS One* 8:e80138. 10.1371/journal.pone.0080138 24224042 PMC3818243

[B17] Elder G. A. Dorr N. P. De Gasperi R. Gama Sosa M. A. Shaughness M. C. Maudlin-Jeronimo E. (2012). Blast exposure induces post-traumatic stress disorder-related traits in a rat model of mild traumatic brain injury. *J. Neurotrauma* 29 2564–2575. 10.1089/neu.2012.2510 22780833 PMC3495123

[B18] Gallo F. T. Katche C. Morici J. F. Medina J. H. Weisstaub N. V. (2018). Immediate early genes, memory and psychiatric disorders: focus on c-Fos, Egr1 and Arc. *Front. Behav. Neurosci.* 12:79. 10.3389/fnbeh.2018.00079 29755331 PMC5932360

[B19] Gama Sosa M. A. De Gasperi R. Janssen P. L. Yuk F. J. Anazodo P. C. Pricop P. E. (2014). Selective vulnerability of the cerebral vasculature to blast injury in a rat model of mild traumatic brain injury. *Acta Neuropathol. Commun.* 2:67. 10.1186/2051-5960-2-67 24938728 PMC4229875

[B20] Gao C. Leng Y. Ma J. Rooke V. Rodriguez-Gonzalez S. Ramakrishnan C. (2020). Two genetically, anatomically and functionally distinct cell types segregate across anteroposterior axis of paraventricular thalamus. *Nat. Neurosci.* 23 217–228. 10.1038/s41593-019-0572-3 31932767 PMC7007348

[B21] Heldt S. A. Elberger A. J. Deng Y. Guley N. H. Del Mar N. Rogers J. (2014). A novel closed-head model of mild traumatic brain injury caused by primary overpressure blast to the cranium produces sustained emotional deficits in mice. *Front. Neurol.* 5:2. 10.3389/fneur.2014.00002 24478749 PMC3898331

[B22] Heyburn L. Abutarboush R. Goodrich S. Urioste R. Batuure A. Statz J. (2019). Repeated low-level blast overpressure leads to endovascular disruption and alterations in TDP-43 and Piezo2 in a rat model of blast TBI. *Front. Neurol.* 10:766. 10.3389/fneur.2019.00766 31417481 PMC6682625

[B23] Heyburn L. Abutarboush R. Goodrich S. Urioste R. Batuure A. Wheel J. (2021). Repeated low-level blast acutely alters brain cytokines, neurovascular proteins, mechanotransduction, and neurodegenerative markers in a rat model. *Front. Cell. Neurosci.* 15:636707. 10.3389/fncel.2021.636707 33679327 PMC7933446

[B24] Hiraoka K. Motomura K. Yanagida S. Ohashi A. Ishisaka-Furuno N. Kanba S. (2017). Pattern of c-Fos expression induced by tail suspension test in the mouse brain. *Heliyon* 3:e00316. 10.1016/j.heliyon.2017.e00316 28616594 PMC5458762

[B25] Hubbard W. B. Hall C. Siva Sai Suijith Sajja V. Lavik E. Vandevord P. (2014). Examining lethality risk for rodent studies of primary blast lung injury. *Biomed. Sci. Instrum.* 50 92–99. 25405409 PMC4591070

[B26] Huber B. R. Meabon J. S. Hoffer Z. S. Zhang J. Hoekstra J. G. Pagulayan K. F. (2016). Blast exposure causes dynamic microglial/macrophage responses and microdomains of brain microvessel dysfunction. *Neuroscience* 319 206–220. 10.1016/j.neuroscience.2016.01.022 26777891 PMC5274718

[B27] Hue C. D. Cho F. S. Cao S. Dale Bass C. R. Meaney D. F. Morrison B. III (2015). Dexamethasone potentiates *in vitro* blood-brain barrier recovery after primary blast injury by glucocorticoid receptor-mediated upregulation of ZO-1 tight junction protein. *J. Cereb. Blood Flow Metab.* 35 1191–1198. 10.1038/jcbfm.2015.38 25757751 PMC4640274

[B28] Ishii H. Onodera M. Ohara S. Tsutsui K.-I. Iijima T. (2018). Sex differences in risk preference and c-Fos expression in paraventricular thalamic nucleus of rats during gambling task. *Front. Behav. Neurosci.* 12:68. 10.3389/fnbeh.2018.00068 29692713 PMC5902494

[B29] Jaiswal S. Knutsen A. K. Wilson C. M. Fu A. H. Tucker L. B. Kim Y. (2019). Mild traumatic brain injury induced by primary blast overpressure produces dynamic regional changes in [18F] FDG uptake. *Brain Res.* 1723:146400. 10.1016/j.brainres.2019.146400 31445032

[B30] Kato T. M. Fujimori-Tonou N. Mizukami H. Ozawa K. Fujisawa S. Kato T. (2019). Presynaptic dysregulation of the paraventricular thalamic nucleus causes depression-like behavior. *Sci. Rep.* 9:16506. 10.1038/s41598-019-52984-y 31712646 PMC6848207

[B31] Kirouac G. J. (2015). Placing the paraventricular nucleus of the thalamus within the brain circuits that control behavior. *Neurosci. Biobehav. Rev.* 56 315–329. 10.1016/j.neubiorev.2015.08.005 26255593

[B32] Koliatsos V. E. Cernak I. Xu L. Song Y. Savonenko A. Crain B. J. (2011). A mouse model of blast injury to brain: initial pathological, neuropathological, and behavioral characterization. *J. Neuropathol. Exp. Neurol.* 70 399–416. 10.1097/NEN.0b013e3182189f06 21487304

[B33] Kontos A. P. Kotwal R. S. Elbin R. Lutz R. H. Forsten R. D. Benson P. J. (2013). Residual effects of combat-related mild traumatic brain injury. *J. Neurotrauma* 30 680–686. 10.1089/neu.2012.2506 23031200

[B34] Kostelnik C. Lucki I. Choi K. H. Browne C. A. (2021). Translational relevance of fear conditioning in rodent models of mild traumatic brain injury. *Neurosci. Biobehav. Rev.* 127 365–376. 10.1016/j.neubiorev.2021.04.037 33961927

[B35] Lister R. G. (1990). Ethologically-based animal models of anxiety disorders. *Pharmacol. Ther.* 46 321–340. 10.1016/0163-7258(90)90021-s 2188266

[B36] Ma J. Du Hoffmann J. Kindel M. Beas B. S. Chudasama Y. Penzo M. A. (2021). Divergent projections of the paraventricular nucleus of the thalamus mediate the selection of passive and active defensive behaviors. *Nat. Neurosci.* 24 1429–1440. 10.1038/s41593-021-00912-7 34413514 PMC8484052

[B37] Mac Donald C. L. Barber J. Jordan M. Johnson A. M. Dikmen S. Fann J. R. (2017). Early clinical predictors of 5-year outcome after concussive blast traumatic brain injury. *JAMA Neurol.* 74 821–829. 10.1001/jamaneurol.2017.0143 28459953 PMC5732492

[B38] Matthews S. C. Strigo I. A. Simmons A. N. O’connell R. M. Reinhardt L. E. Moseley S. A. (2011). A multimodal imaging study in US veterans of Operations Iraqi and Enduring Freedom with and without major depression after blast-related concussion. *Neuroimage* 54 S69–S75. 10.1016/j.neuroimage.2010.04.269 20451622

[B39] McCabe J. T. Tucker L. B. (2020). Sex as a biological variable in preclinical modeling of blast-related traumatic brain injury. *Front. Neurol.* 11:541050. 10.3389/fneur.2020.541050 33101170 PMC7554632

[B40] Morgan M. Pfaff D. (2002). Estrogen’s effects on activity, anxiety, and fear in two mouse strains. *Behav. Brain Res.* 132 85–93. 10.1016/s0166-4328(01)00398-9 11853861

[B41] Needham C. E. Ritzel D. Rule G. T. Wiri S. Young L. (2015). Blast testing issues and TBI: experimental models that lead to wrong conclusions. *Front. Neurol.* 6:72. 10.3389/fneur.2015.00072 25904891 PMC4389725

[B42] Nestler E. J. Hyman S. E. (2010). Animal models of neuropsychiatric disorders. *Nat. Neurosci.* 13:1161. 10.1093/ijnp/pyac024 20877280 PMC3750731

[B43] Nonaka M. Taylor W. W. Bukalo O. Tucker L. B. Fu A. H. Kim Y. (2021). Behavioral and myelin-related abnormalities after blast-induced mild traumatic brain injury in mice. *J. Neurotrauma* 38 1551–1571. 10.1089/neu.2020.7254 33605175 PMC8126426

[B44] Ogawa S. Chan J. Gustafsson J. A. Korach K. S. Pfaff D. W. (2003). Estrogen increases locomotor activity in mice through estrogen receptor α: specificity for the type of activity. *Endocrinology* 144 230–239. 10.1210/en.2002-220519 12488349

[B45] Ou Y. Clifton B. A. Li J. Sandlin D. Li N. Wu L. (2022). Traumatic brain injury induced by exposure to blast overpressure via ear canal. *Neural Regen. Res.* 17:115. 10.4103/1673-5374.314311 34100446 PMC8451570

[B46] Owens B. D. Kragh J. F. Jr. Wenke J. C. Macaitis J. Wade C. E. Holcomb J. B. (2008). Combat wounds in operation Iraqi Freedom and operation Enduring Freedom. *J. Trauma Acute Care Surg.* 64 295–299. 10.1097/ta.0b013e318163b875 18301189

[B47] Palanza P. Gioiosa L. Parmigiani S. (2001). Social stress in mice: gender differences and effects of estrous cycle and social dominance. *Physiol. Behav.* 73 411–420. 10.1016/s0031-9384(01)00494-2 11438369

[B48] Park K. Chung C. (2019). Systemic cellular activation mapping of an extinction-impaired animal model. *Front. Cell. Neurosci.* 13:99. 10.3389/fncel.2019.00099 30941016 PMC6433791

[B49] Pellow S. Chopin P. File S. E. Briley M. (1985). Validation of open: closed arm entries in an elevated plus-maze as a measure of anxiety in the rat. *J. Neurosci. Methods* 14 149–167. 10.1016/0165-0270(85)90031-7 2864480

[B50] Penzo M. A. Robert V. Tucciarone J. De Bundel D. Wang M. Van Aelst L. (2015). The paraventricular thalamus controls a central amygdala fear circuit. *Nature* 519 455–459. 10.1038/nature13978 25600269 PMC4376633

[B51] Perez Garcia G. Perez G. M. De Gasperi R. Gama Sosa M. A. Otero-Pagan A. Pryor D. (2021). Progressive cognitive and post-traumatic stress disorder-related behavioral traits in rats exposed to repetitive low-level blast. *J. Neurotrauma* 38 2030–2045. 10.1089/neu.2020.7398 33115338 PMC8418528

[B52] Perez-Garcia G. Sosa M. a. G. De Gasperi R. Tschiffely A. E. Mccarron R. M. Hof P. R. (2019). Blast-induced” PTSD”: evidence from an animal model. *Neuropharmacology* 145 220–229. 10.1016/j.neuropharm.2018.09.023 30227150 PMC12199260

[B53] Porsolt R. D. Le Pichon M. Jalfre M. (1977). Depression: a new animal model sensitive to antidepressant treatments. *Nature* 266 730–732. 10.1038/266730a0 559941

[B54] Raghupathi R. Mcintosh T. K. Smith D. H. (1995). Cellular responses to experimental brain injury. *Brain Pathol.* 5 437–442. 10.1111/j.1750-3639.1995.tb00622.x 8974626

[B55] Ratliff W. A. Mervis R. F. Citron B. A. Schwartz B. Rubovitch V. Schreiber S. (2019). Mild blast-related TBI in a mouse model alters amygdalar neurostructure and circuitry. *Exp. Neurol.* 315 9–14. 10.1016/j.expneurol.2019.01.020 30711646 PMC6622172

[B56] Ratliff W. A. Mervis R. F. Citron B. A. Schwartz B. Rubovitch V. Schreiber S. (2020). Effect of mild blast-induced TBI on dendritic architecture of the cortex and hippocampus in the mouse. *Sci. Rep.* 10:2206. 10.1038/s41598-020-59252-4 32042033 PMC7010659

[B57] Rex T. Elberger A. Deng Y. Guley N. Hines-Beard J. D’surney L. (2013). Visual deficits in mice after mild traumatic brain injury produced using a novel closed-head model of primary overpressure blast. *Invest. Ophthalmol. Vis. Sci.* 54 5095–5095. 24478749

[B58] Rodriguez U. A. Zeng Y. Parsley M. A. Hawkins B. E. Prough D. S. Dewitt D. S. (2019). Effects of blast-induced neurotrauma on pressurized rodent middle cerebral arteries. *J. Vis. Exp.* 146:e58792. 10.3791/58792 30985764

[B59] Rosenfeld J. V. Ford N. L. (2010). Bomb blast, mild traumatic brain injury and psychiatric morbidity: a review. *Injury* 41 437–443. 10.1016/j.injury.2009.11.018 20189170

[B60] Rowson S. A. Pleil K. E. (2021). Influences of stress and sex on the paraventricular thalamus: implications for motivated behavior. *Front. Behav. Neurosci.* 15:636203. 10.3389/fnbeh.2021.636203 33716683 PMC7953143

[B61] Rubovitch V. Ten-Bosch M. Zohar O. Harrison C. R. Tempel-Brami C. Stein E. (2011). A mouse model of blast-induced mild traumatic brain injury. *Exp. Neurol.* 232 280–289. 21946269 10.1016/j.expneurol.2011.09.018 PMC3202080

[B62] Russell A. L. Richardson M. R. Bauman B. M. Hernandez I. M. Saperstein S. Handa R. J. (2018b). Differential responses of the HPA axis to mild blast traumatic brain injury in male and female mice. *Endocrinology* 159 2363–2375. 10.1210/en.2018-00203 29701827

[B63] Russell A. L. Handa R. J. Wu T. J. (2018a). Sex-dependent effects of mild blast-induced traumatic brain injury on corticotropin-releasing factor receptor gene expression: potential link to anxiety-like behaviors. *Neuroscience* 392 1–12. 10.1016/j.neuroscience.2018.09.014 30248435

[B64] Ryan-Gonzalez C. Kimbrel N. A. Meyer E. C. Gordon E. M. Debeer B. B. Gulliver S. B. (2019). Differences in post-traumatic stress disorder symptoms among post-9/11 veterans with blast-and non-blast mild traumatic brain injury. *J. Neurotrauma* 36 1584–1590. 10.1089/neu.2017.5590 30511882

[B65] Sajja V. S. S. S. Hubbard W. B. Vandevord P. J. (2015). Subacute oxidative stress and glial reactivity in the amygdala are associated with increased anxiety following blast neurotrauma. *Shock* 44 71–78. 10.1097/SHK.0000000000000311 25521536

[B66] Säljö A. Bao F. Shi J. Hamberger A. Hansson H. A. Haglid K. G. (2002). Expression of c-Fos and c-Myc and deposition of β-APP in neurons in the adult rat brain as a result of exposure to short-lasting impulse noise. *J. Neurotrauma* 19 379–385. 10.1089/089771502753594945 11939505

[B67] Saunders N. R. Dziegielewska K. M. Møllgård K. Habgood M. D. (2015). Markers for blood-brain barrier integrity: how appropriate is Evans blue in the twenty-first century and what are the alternatives? *Front. Neurosci.* 9:385. 10.3389/fnins.2015.00385 26578854 PMC4624851

[B68] Sawyer T. W. Wang Y. Ritzel D. V. Josey T. Villanueva M. Shei Y. (2016). High-fidelity simulation of primary blast: direct effects on the head. *J. Neurotrauma* 33 1181–1193. 10.1089/neu.2015.3914 26582146

[B69] Schindler A. G. Terry G. E. Wolden-Hanson T. Cline M. Park M. Lee J. (2021). Repetitive blast promotes chronic aversion to neutral cues encountered in the peri-blast environment. *J. Neurotrauma* 38 940–948. 10.1089/neu.2020.7061 33138684 PMC9208723

[B70] Shepherd J. K. Grewal S. S. Fletcher A. Bill D. J. Dourish C. T. (1994). Behavioural and pharmacological characterisation of the elevated “zero-maze” as an animal model of anxiety. *Psychopharmacology* 116 56–64. 10.1007/BF02244871 7862931

[B71] Shively S. B. Horkayne-Szakaly I. Jones R. V. Kelly J. P. Armstrong R. C. Perl D. P. (2016). Characterisation of interface astroglial scarring in the human brain after blast exposure: a post-mortem case series. *Lancet Neurol.* 15 944–953. 10.1016/S1474-4422(16)30057-6 27291520

[B72] Stemper B. D. Shah A. S. Budde M. D. Olsen C. M. Glavaski-Joksimovic A. Kurpad S. N. (2016). Behavioral outcomes differ between rotational acceleration and blast mechanisms of mild traumatic brain injury. *Front. Neurol.* 7:31. 10.3389/fneur.2016.00031 27014184 PMC4789366

[B73] Tucker L. B. McCabe J. T. (2017). Behavior of male and female C57BL/6J mice is more consistent with repeated trials in the elevated zero maze than in the elevated plus maze. *Front. Behav. Neurosci.* 11:13. 10.3389/fnbeh.2017.00013 28184191 PMC5266707

[B74] Tucker L. B. McCabe J. T. (2021). Measuring anxiety-like behaviors in rodent models of traumatic brain injury. *Front. Behav. Neurosci.* 15:682935. 10.3389/fnbeh.2021.682935 34776887 PMC8586518

[B75] Tucker L. B. Burke J. F. Fu A. H. Mccabe J. T. (2017). Neuropsychiatric symptom modeling in male and female C57BL/6J mice after experimental traumatic brain injury. *J. Neurotrauma* 34 890–905. 10.1089/neu.2016.4508 27149139 PMC5314988

[B76] Tucker L. B. Fu A. H. McCabe J. T. (2016). Performance of male and female C57BL/6J mice on motor and cognitive tasks commonly used in pre-clinical traumatic brain injury research. *J. Neurotrauma* 33 880–894. 10.1089/neu.2015.3977 25951234 PMC4860656

[B77] Tucker L. B. Velosky A. G. Fu A. H. McCabe J. T. (2019). Chronic neurobehavioral sex differences in a murine model of repetitive concussive brain injury. *Front. Neurol.* 10:509. 10.3389/fneur.2019.00509 31178814 PMC6538769

[B78] Unnikrishnan G. Mao H. Sajja V. Van Albert S. Sundaramurthy A. Rubio J. E. (2021). Animal orientation affects brain biomechanical responses to blast-wave exposure. *J. Biomech. Eng.* 143:051007. 10.1115/1.4049889 33493319

[B79] Vu P. A. Mcnamara E. H. Liu J. Tucker L. B. Fu A. H. McCabe J. T. (2021). Behavioral responses following repeated bilateral frontal region closed head impacts and fear conditioning in male and female mice. *Brain Res.* 1750:147147. 10.1016/j.brainres.2020.147147 33091394

[B80] Vu P. A. Tucker L. B. Liu J. Mcnamara E. H. Tran T. Fu A. H. (2018). Transient disruption of mouse home cage activities and assessment of orexin immunoreactivity following concussive-or blast-induced brain injury. *Brain Res.* 1700 138–151. 10.1016/j.brainres.2018.08.034 30176241

[B81] Wagner A. K. Willard L. A. Kline A. E. Wenger M. K. Bolinger B. D. Ren D. (2004). Evaluation of estrous cycle stage and gender on behavioral outcome after experimental traumatic brain injury. *Brain Res.* 998 113–121. 10.1016/j.brainres.2003.11.027 14725974

[B82] Walker W. C. Franke L. M. Mcdonald S. D. Sima A. P. Keyser-Marcus L. (2015). Prevalence of mental health conditions after military blast exposure, their co-occurrence, and their relation to mild traumatic brain injury. *Brain Inj.* 29 1581–1588. 10.3109/02699052.2015.1075151 26479126

[B83] Weng J. C. Wu S. K. Lin W. L. Tseng W. Y. (2011). Detecting blood-brain barrier disruption within minimal hemorrhage following transcranial focused ultrasound: a correlation study with contrast-enhanced MRI. *Magn. Reson. Med.* 65 802–811. 10.1002/mrm.22643 20941741

[B84] Xie K. Kuang H. Tsien J. Z. (2013). Mild blast events alter anxiety, memory, and neural activity patterns in the anterior cingulate cortex. *PLoS One* 8:e64907. 10.1371/journal.pone.0064907 23741416 PMC3669016

[B85] Yanagida S. Motomura K. Ohashi A. Hiraoka K. Miura T. Kanba S. (2016). Effect of acute imipramine administration on the pattern of forced swim-induced c-Fos expression in the mouse brain. *Neurosci. Lett.* 629 119–124. 10.1016/j.neulet.2016.06.059 27373591

[B86] Yang F. Y. Huang S. F. Cheng I. H. (2016). Behavioral alterations following blood-brain barrier disruption stimulated by focused ultrasound. *Oncotarget* 7 27916–27925. 10.18632/oncotarget.8444 27034007 PMC5053698

